# Frailty is associated with lower-limb osteoarthritis incidence over six-years regardless of sex and type of frailty index in the Canadian longitudinal study on aging

**DOI:** 10.1016/j.ocarto.2026.100827

**Published:** 2026-05-27

**Authors:** Carson Halliwell, Rebecca Moyer, Alexandra Legge, Olga Theou, Mathieu Bélanger, Said Mekari, Myles W. O'Brien

**Affiliations:** a Centre de Formation Médicale du Nouveau-Brunswick, Moncton, NB, Canada; b Faculty of Medicine and Health Science, Université de Sherbrooke, Sherbrooke, QC, Canada; c Faculty of Health, Dalhousie University, Halifax, NS, Canada; d Faculty of Medicine, Dalhousie University, Halifax, NS, Canada

**Keywords:** Aging, Musculoskeletal health, Epidemiology, Health deficits, CLSA

## Abstract

**Objective:**

We aimed to test whether increases in frailty are associated with a higher likelihood of developing lower-limb osteoarthritis over six-years, with stronger associations in females, and to compare whether results are specific to a self-reported versus a comprehensive frailty index.

**Design:**

Data were drawn from the Canadian Longitudinal Study on Aging's comprehensive cohort. Frailty was quantified using a self-reported (FI-SELF; 46-items; n = 24,212) and comprehensive frailty index (FI-COM; 86-items; n = 13,757) at baseline, three-year, and six-year follow-up. Incident osteoarthritis was defined as self-reporting whether a doctor had ever diagnosed hip or knee osteoarthritis. Time-varying Cox proportional hazards models examined the relation between frailty and osteoarthritis incidence. Models included a frailty × sex interaction and adjusted for age, body mass index, and marital status. Sex-stratified models were also conducted.

**Results:**

Higher frailty was associated with a greater likelihood of developing lower-limb osteoarthritis over six-years. Using the FI-SELF, each 0.01-point increase (one additional health deficit) corresponded to a 4.7% higher hazard of osteoarthritis in males (HR = 1.047, 95% CI 1.042–1.051) and a 5.0% higher hazard in females (HR = 1.050, 95% CI 1.047–1.054). However, there was no significant frailty × sex interaction for either the FI-SELF (p = 0.057) or FI-COM (p = 0.406), indicating similar associations across sexes. Findings were consistent between the FI-SELF and FI-COM. Females exhibited higher frailty scores than males at baseline, three-year, and six-year follow-up (all p < 0.001).

**Conclusion:**

Longitudinal changes in frailty are independently associated with incident lower-limb osteoarthritis in both males and females over six-years, with similar results when using either the FI-SELF or FI-COM.

## 1 Introduction

One of the most problematic expressions of population aging is frailty, and the increasing number of older adults with multiple health challenges [1]. Frailty can be considered a state of increased vulnerability to poor resolution of homeostasis after a stressor event, which increases the risk of adverse health outcomes, including falls, delirium, and disability [2]. Frailty develops because of age-related decline across multiple physiological systems. A decline in musculoskeletal health is particularly prominent with aging and higher frailty levels [3]. Given the central role of frailty in the process of musculoskeletal decline, understanding the relation between frailty and conditions such as osteoarthritis is important for informing prevention and early intervention strategies.

Musculoskeletal health is essential for independence, continued ambulatory function, and participation in society [4]. Osteoarthritis is one of the largest contributors of disability adjusted life years among non-communicable disease worldwide [5], with knee and hip osteoarthritis making up over 80% of cases [6], and its impact continues to rise exponentially [7]. An estimated 87% of individuals with osteoarthritis have at least one other comorbidity such as cardiovascular disease, dementia, or rheumatic disease [8,9], ultimately contributing to the high rates of frailty ranging from 24 to 60% [[10], [11], [12], [13]] in this population. Evidence shows a clear link between frailty and osteoarthritis, with results demonstrating that individuals with osteoarthritis are at an increased risk of developing frailty [14], higher frailty is associated with worse long-term patient reported outcomes, and increased odds of total joint replacement [15]. Further, frailty has been shown to moderate the relation between radiographic knee osteoarthritis severity and pain, where individuals with higher frailty levels report worse pain compared to those with low frailty with the same radiographic severity level [16].

Although frailty and osteoarthritis share common age-related mechanisms [16,17], including inflammation, reduced physical activity, and declines in physiological reserve, they are considered distinct clinical constructs. Osteoarthritis primarily reflects joint pathology, whereas frailty reflects a broader state of multisystem vulnerability resulting from the accumulation of deficits across multiple physiological systems [2]. Importantly, frailty can occur in the absence of osteoarthritis, and many individuals with osteoarthritis are not frail, suggesting that frailty may represent a systemic factor rather than simply a manifestation of osteoarthritis. Despite these growing insights, the majority of research focuses on how frailty impacts osteoarthritis, and how osteoarthritis leads to frailty [14]. Previous research from Misra and colleagues (2014) has linked osteoarthritis with an increased risk of physical frailty [14]; however, there is there is a marked lack of literature examining relations between multisystem frailty and osteoarthritis. An emerging theory exists that frailty may be the precursor expediting the osteoarthritis process [17]. Potential mechanisms linking frailty and osteoarthritis include reduced physiological reserve, lower physical activity, and chronic low-grade inflammation [17]. These factors have each been associated with osteoarthritis development and may contribute to osteoarthritis onset over time [[18], [19], [20], [21]]. Longitudinal research is needed to better characterize the relation between changes in frailty and osteoarthritis onset, and to determine whether worsening frailty precedes the development of osteoarthritis over time.

A critical but understudied factor in the frailty-osteoarthritis relation are sex differences. Females are at approximately twice the risk of developing osteoarthritis in their lifetime compared to males, tend to be more severe, have worse physical function, and increased pain compared to males [22]. Similarly, females tend to have worse levels of frailty compared to age matched males [23] and nearly double the prevalence of males [24]. Importantly, studies vary widely in how frailty is measured. Most commonly, frailty is measured using a self-report frailty index [25] to more comprehensive assessments that include self-report measures, as well as cognitive testing, spirometry, electrocardiogram abnormalities, and physical examination findings [16,25,26]. These methodological differences may obscure true sex-specific patterns, as self-report indices may capture different aspects of vulnerability than objectively measured health outcomes. Addressing the gaps created by the use of cross-sectional study designs, not accounting for sex differences and using distinct frailty measurement approaches could support more precise, sex-specific strategies for managing frailty and osteoarthritis.

Frailty and osteoarthritis are interrelated conditions that frequently coexist. Frailty may act as a marker of vulnerability associated with osteoarthritis risk, rather than a direct causal factor, reflecting the concept that “the sick are more likely to get sicker”. With an aging population world-wide, the overlap between frailty and osteoarthritis is becoming increasingly prevalent. Therefore, the purpose of the current study was to test the hypothesis that increases in frailty will be associated with a higher likelihood of developing lower-limb osteoarthritis over six-years and this relation will be stronger in females compared to males. A secondary aim was to compare results obtained when using a self-reported frailty index versus a comprehensive frailty index incorporating both self-reported and clinical measures.

## 2 Methods

*Canadian Longitudinal Study on Aging (CLSA).* Data for this study were obtained exclusively from the comprehensive cohort of the CLSA, a large, population-based study examining the biological, medical, psychological, and social determinants of healthy aging among Canadians aged 45–85 years [27]. The comprehensive cohort consists of approximately 30,000 participants recruited from seven Canadian provinces who lived within a 25-50-km radius of one of 11 data collection sites [28]. Participants were identified using Provincial Health Registration Databases and random digit dialing [28]. Each participant completed an in-home interview followed by standardized assessments and biological sample collection at a CLSA data collection site [28]. Participants were reassessed every three years. Baseline data (collected from 2011 to 2015) and follow-up data from the first (2015–2018) and second (2018–2021) waves were available at the time of analysis.

Individuals were excluded from the CLSA if they were unable to communicate in English or French, resided in the three territories, lived on federal First Nations reserves, were full-time members of the Canadian Armed Forces, had cognitive impairment at the time of recruitment, or were institutionalized (e.g., living in long-term care facilities) [27]. The protocol of this study was approved by the CLSA Data and Sample Access Committee. This project received ethical approval from the Université de Sherbrooke ethics review board (REB: 2025–4914). Data for this study were obtained through formal application to the CLSA, whose standard operating and consent procedures are publicly available (https://www.clsa-elcv.ca/). All CLSA participants provided informed consent at enrollment, and researchers accessed only deidentified data for analysis. All participants self-reported age, sex and marital status. Body mass index (BMI) was calculated as mass (kg) divided by height squared (m^2^).

*Frailty Index.* The current study used self-report (FI-SELF) and comprehensive (FI-COM) frailty indices. The FI-SELF is derived exclusively from self-reported health information, capturing deficits across domains such as chronic conditions, activities of daily living, mental health, and self-rated health. In contrast, the FI-COM is a broader index that integrates self-reported data with clinical, laboratory, and objectively measured variables, including cognition, spirometry, cardiovascular measures, and sensory function. While the FI-COM provides a more comprehensive assessment of physiological and functional health, the FI-SELF may be more readily applicable in clinical and large-scale population settings, as it relies solely on information that can be obtained without specialized testing or in-person assessments. A detailed description of their original calculation and validation has been published previously [25]. Both the FI-SELF and FI-COM used in the current study were based on the deficit accumulation model [29], originally developed and validated using the CLSA dataset [25] following established protocols [30]. The initial FI-SELF included 48 items, following standard practice [26], two items relating to osteoarthritis were removed so our exposure (frailty) did not directly overlap with the outcome (osteoarthritis), leaving a total of 46-items (Supplemental Table 1), each scored as 0 (no deficit) or 1 (deficit). Interval or ordinal variables were coded proportionally (e.g., self-rated health: excellent = 0, very good = 0.25, good = 0.5, fair = 0.75, poor = 1). Only participants with data for at least 80% [30] of the FI-SELF items at all timepoints were included (n = 24,212). The generated FI-SELF includes themes such as chronic conditions, self-rated health, activities of daily living, and mental health. The initial FI-COM included 118 items. Any items related to osteoarthritis were removed (4 osteoarthritis items) and any items that were not included at each timepoint were also removed (28 items) yielding a total of 86 items remaining. Due to COVID-19 public health restrictions during collections happening between 2018 and 2021, 10,664 participants did not have in person measurements and were excluded from the FI-COM calculation. Participants were included in the current analyses if they had data for a minimum of 80% [30] of the FI-COM items across all timepoints (n = 13,757). The generated FI-COM included themes such as chronic conditions, self-rated health, activities of daily living, mental health, cognition, spirometry, hearing and vision, and cardiac outcomes (Supplemental Table 2). Despite these exclusions, both frailty indices still include over 30 deficits, ensuring reliable calculation [30]. The indices were calculated by dividing the number of deficits by the total number of items assessed for each participant (e.g., 4/46 = 0.09), with higher values (closer to 1.00) indicating greater frailty. All frailty indices were calculated at baseline, three- and six-year follow-up.

*Lower-Limb Osteoarthritis*. The presence of lower-limb osteoarthritis (hip or knee) was the primary outcome. The presence of this condition was based on participants self-reporting whether a doctor had ever told them that they have osteoarthritis. For example, participants were asked, “Has a doctor ever told you that you have knee osteoarthritis?” and “Has a doctor ever told you that you have hip osteoarthritis?“. Participants who reported having either hip or knee osteoarthritis were coded as 1. Incident knee and hip osteoarthritis was considered if participants reported “no” at baseline and “yes” at three or six-year follow-up. Exploratory analyses were also run on hip and knee osteoarthritis separately.

*Statistical Analyses*. Participant characteristics were stratified by sex. Descriptive comparisons between males and females were performed using independent samples *t*-tests for continuous variables or Chi-square for categorical data for both FI-SELF and FI-COM groups. Normality was satisfied based on the central limit theorem [31]. Differences in frailty and osteoarthritis presence were compared between males and females using a 3 × 2 (three time points, two groups) mixed methods analysis of variance model. Cox proportional hazards models with time-varying covariates were used to examine how changes in frailty over time influenced the risk of developing lower-limb osteoarthritis. The model included a frailty × sex interaction to examine whether frailty increased osteoarthritis risk differently in males vs females. Regardless of a significant frailty × sex interaction, all analyses were also conducted separately for males and females due to previously established frailty and osteoarthritis differences by sex [22,23]. Models were adjusted for covariates including baseline age and marital status; BMI (continuous) was included as a time varying co-variate. Variables that are included in the frailty index were not included as a covariate. Frailty was modeled as a time-varying covariate to allow the hazard ratios to reflect the dynamic influence of frailty changes at baseline, three-, and six-year follow-up. A HR greater than 1 indicated an increased risk of developing osteoarthritis with higher frailty, whereas a HR less than 1 indicated a decreased risk. Frailty values were multiplied by 100 so that hazard ratios represent a 0.01-unit increase in frailty rather than a 1.00-unit increase. This approach was used because a 1.00-unit change is not physiologically possible, while a 0.01-unit change reflects the accumulation of approximately one health deficit [26]. All analyses were completed using both the FI-SELF and FI-COM. A clinically important frailty progression was defined as a 0.03 point increase in the frailty index [32]. To assess the robustness of findings, sensitivity analyses were performed by restricting the self-reported frailty index cohort (n = 24,212) to participants with available comprehensive frailty index data (n = 13,757). Identical Cox proportional hazards models were completed in this restricted sample. All statistical analyses were performed using R-studio software (version 2025.05.1 + 513; R Foundation for Statistical Computing, Vienna, Austria).

## 3 Results

**Fig. 1:**
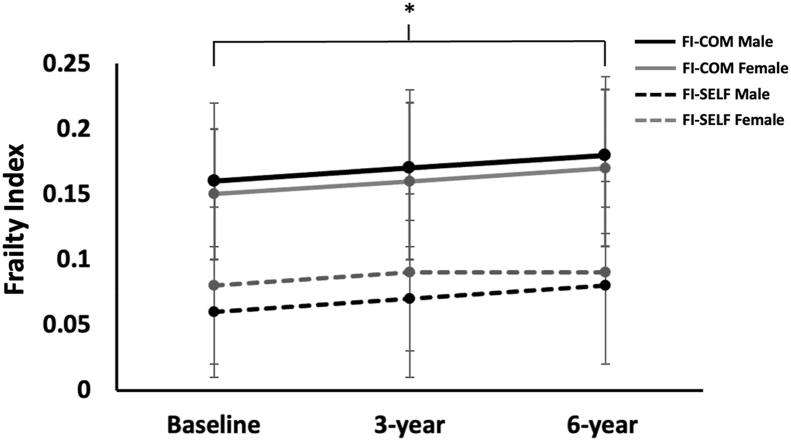
Change in frailty using the FI-SELF (dashed line) and FI-COM (solid line), over baseline, three-year and six-year follow-up for males (black) and females (grey). Error bars represent standard deviation. Asterisks (∗) represent statistical difference over six-years (p < 0.001).

*Participant Characteristics*. Baseline participant characteristics are presented in Table 1. Using the FI-SELF, the final sample included 11,791 males (mean age 62.2 ± 9.8 years) and 12,421 females (mean age 61.9 ± 9.7 years); however, females had higher frailty than males at baseline (0.08 ± 0.06 vs. 0.06 ± 0.05, p < 0.001), three-year (0.09 ± 0.06 vs. 0.07 ± 0.06, p < 0.001), and six-year follow-up (0.09 ± 0.07 vs. 0.08 ± 0.06, p < 0.001) (Fig. 1). Using the FI-COM, the final sample included 6543 males (mean age 62.5 ± 9.5 years) and 7214 females (mean age 61.6 ± 9.5 years). When using the FI-COM, females had lower frailty than males at baseline (0.15 ± 0.06 vs. 0.16 ± 0.05, p < 0.001), three-year (0.16 ± 0.6 vs. 0.17 ± 0.06, p < 0.001), and six-year follow-up (0.17 ± 0.06 vs. 0.18 ± 0.06, p < 0.001) (Fig. 1).

**Table 1:** Participants’ demographics and clinical characteristics at baseline

Self-Reported Frailty	*Pooled Sample*	*Males*	*Females*	*Males vs Females*
n	24,212	11,791	12,421	–
Age (years)	62.0 ± 9.8	62.2 ± 9.8	61.9 ± 9.7	p = 0.89
BMI (kg/m^2^)	27.8 ± 5.4	27.9 ± 5.4	28.3 ± 4.7	p < 0.001
Ethnicity, no of white (%)	22,691 (93.7%)	11,040 (93.8%)	11,651 (93.6%)	–
Frailty index	0.07 ± 0.05	0.08 ± 0.05	0.07 ± 0.05	p < 0.001
Hip osteoarthritis, no. of (%)	1930 (8.0%)	670 (5.7%)	1260 (10.1)	p < 0.001
Knee osteoarthritis, no. of (%)	3543 (14.6%)	1380 (11.8%)	2163 (17.4)	p < 0.001
Married, no. of (%)	17,126 (70.7%)	9409 (79.8%)	7717 (62.1%)	p < 0.001

**Comprehensive frailty**		*Males*	*Females*	

n	13,757	6543	7214	–
Age (years)	62.0 ± 9.5	62.5 ± 9.5	61.6 ± 9.5	p < 0.001
BMI (kg/m^2^)	27.9 ± 5.3	28.2 ± 4.6	27.6 ± 5.8	p < 0.001
Ethnicity, no of white (%)	13,066 (95.0%)	6201 (94.8%)	6865 (95.2%)	–
Frailty index	0.16 ± 0.05	0.16 ± 0.05	0.15 ± 0.06	p < 0.001
Hip osteoarthritis, no. of (%)	1085 (7.9%)	372 (5.7%)	713 (9.9%)	p < 0.001
Knee osteoarthritis, no. of (%)	1996 (14.5%)	781 (11.9%)	1215 (16.8%)	p < 0.001
Married, no. of (%)	9945 (72.2%)	5301 (81.0%)	4644 (64.4%)	p < 0.001

Values represent means ± standard deviations or proportions (%).

Note: BMI = Body Mass Index

**Fig. 2:**
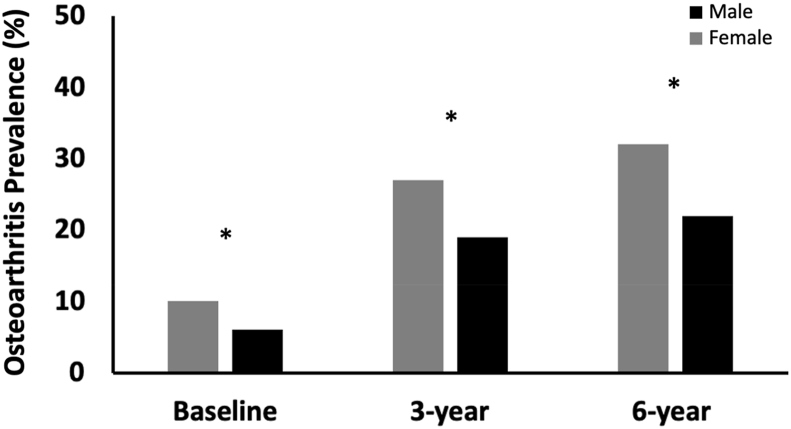
Lower-limb osteoarthritis prevalence in males (black) and females (grey) at baseline, three-year, and six-year follow-up. Asterisks (∗) represent statistical difference between sexes (p < 0.001).

Females consistently had a higher prevalence of both hip and knee osteoarthritis than males at baseline (hip: 9.9% vs. 5.7%, p < 0.001; knee: 16.8% vs. 11.9%, p < 0.001), at three-year (hip: 13.6% vs. 7.6%, p < 0.001; knee: 20.9% vs. 14.9%, p < 0.001), and at six-year follow-up (hip: 14.9% vs. 8.9%, p < 0.001; knee: 24.1% vs. 17.0%, p < 0.001) (Fig. 2). There was no significant frailty × sex interaction for osteoarthritis incidence for either the FI-SELF (p = 0.057) or FI-COM (p = 0.406) (Table 2).

**Table 2:** Cox regression output for the self-reported and comprehensive frailty indices. Data are presented for the full sample, and males and females separately.

Self-Reported Frailty	*Pooled Sample*	*Males*	*Females*
Sex	0.723 [0.680: 0.771]	–	–
Frailty × sex	0.995 [0.990: 1.002]	–	–
Frailty index	1.051 [1.048: 1.054]	1.047 [1.042: 1.051]	1.050 [1.047: 1.054]
Age (years)	1.021 [1.019: 1.023]	1.020 [1.017: 1.023]	1.021 [1.019: 1.024]
BMI (kg/m^2^)	1.000 [0.999: 1.001]	1.000 [1.000: 1.000]	1.000 [1.000: 1.000]
Married, no. of (%)	1.000 [ 1.000: 1.000]	0.958 [0.923: 0.995]	1.000 [1.000:1.000]

**Comprehensive frailty**	*Pooled Sample*	*Males*	*Females*

Sex	0.667 [0.575: 0.774]	–	–
Frailty × sex	0.997 [0.989: 1.004]	–	–
Frailty index	1.049 [1.044: 1.055]	1.047 [1.039: 1.055]	1.049 [1.043: 1.055]
Age (years)	1.016 [1.013: 1.019]	1.015 [1.010: 1.020]	1.016 [1.013: 1.020]
BMI (kg/m^2^)	1.001 [1.001: 1.002]	1.003 [1.002: 1.004]	1.001 [1.000: 1.002]
Married, no. of (%)	1.000 [ 1.000: 1.000]	0.943 [0.895: 0.994]	1.000 [1.000:1.000]

Values represent hazard ratios and [95% confidence intervals], representing the risk of lower-limb osteoarthritis development for every 0.01-point increase in frailty.

*Risk of Osteoarthritis Incidence:* Across both sexes and frailty indices, higher baseline frailty was consistently associated with an increased risk of developing lower-limb osteoarthritis over six-years (p < 0.001). Among males, 3220 (26%) and 2066 (32%) experienced clinically important frailty progression using the FI-SELF and FI-COM, respectively. Each 0.01-point increase in frailty was associated with a 4.7% higher hazard of incident osteoarthritis for both the FI-SELF (HR = 1.047, 95% CI 1.042–1.051) and FI-COM (HR = 1.047, 95% CI 1.039–1.055), independent of age, BMI, and marital status. Similarly, among females, 4049 (32%) and 2338 (32%) showed clinically important frailty progression using the FI-SELF and FI-COM. Each 0.01-point increase in frailty was associated with a 5.0% higher hazard of osteoarthritis using the FI-SELF (HR = 1.050, 95% CI 1.047–1.054) and a 4.9% higher hazard using the FI-COM (HR = 1.049, 95% CI 1.043–1.055), adjusting for the same covariates (Table 2). Results stratified by knee and hip osteoarthritis are provided in Supplemental Table 3. Sensitivity analyses showed similar results in the restricted FI-SELF (Supplemental Table 4).

## 4 Discussion

This study investigated whether increases in frailty were associated with an increased risk of lower-limb osteoarthritis in males and females over six-years using data from the CLSA. Our hypothesis that the relation between frailty and osteoarthritis would be stronger in females compared to males was not supported. Consistent with previous reports, females exhibited approximately 1.5-times the prevalence of osteoarthritis [22]. Supporting our hypothesis, frailty was associated with lower-limb osteoarthritis incidence; however, no difference was noted between sexes after adjusting for age, BMI, and marital status. Specifically, each 0.01-point increase in the frailty index corresponded to a 4.7–5.0% higher risk of developing osteoarthritis. Importantly, these associations were consistent regardless of whether frailty was measured using the FI-SELF or FI-COM, underscoring the robustness of the relation between frailty and osteoarthritis risk. These findings indicate that frailty is longitudinally associated with osteoarthritis development.

This study provides evidence that frailty is associated with incident lower-limb osteoarthritis in both males and females over a six-year period. Whereas most prior research relies on a single baseline frailty score [15,16], implicitly treating frailty as static, our study builds on this by tracking frailty longitudinally, allowing risk estimates to reflect meaningful changes in health status over time. Importantly, both the FI-COM, which incorporates clinical assessments such as physical examinations, spirometry, and electrocardiogram measures, and the brief FI-SELF performed similarly in predicting osteoarthritis risk. Meaning that even modest increases in frailty, regardless of how it is measured, elevate the hazard of developing hip or knee osteoarthritis. This is particularly important because the FI-SELF is far easier and less time-consuming to administer, yet yields comparable risk estimates, offering a highly scalable option for clinical screening. At the same time, consistent with previous research, the FI-COM classified participants as more severely frail, emphasizing that frailty severity should be tailored to the measurement tool utilized [25]. Future research should investigate why the FI-COM identified higher frailty in males while the FI-SELF indicated higher frailty in females, and whether this reflects true physiological differences, or differing self-perceptions of health. Regardless, these findings underscore frailty as a measurable risk factor for lower-limb osteoarthritis, reinforcing its relevance as a key determinant in osteoarthritis development.

These findings have several important clinical implications. In prior frailty research, a 0.03-point increase in the frailty index has been widely considered a clinically meaningful increase in frailty levels [32]. Applying this threshold to the current study, each clinically significant 0.03-point increase in frailty was associated with approximately a 15% higher hazard of developing osteoarthritis in both males and females. To provide context, this magnitude of risk is comparable to other well-established osteoarthritis predictors from a recent systematic review with meta-analysis [33], including a 15% increased hazard per year of age (HR = 1.15), a 17% increased hazard per unit increase in BMI (HR = 1.17), and 2.7-fold increased odds associated with prior knee injury (HR = 2.67) [33]. Based on ∼30% of participants demonstrating a clinically meaningful increase in frailty over six-years, almost 1-in-3 individuals have a 15% increased risk of incident lower-limb osteoarthritis. These comparisons underscore the importance of routinely assessing frailty as part of osteoarthritis evaluations and highlight its potential as a modifiable risk factor for the disease.

Importantly, frailty can be effectively managed using a multidimensional approach, as outlined in the Canadian Frailty Network's AVOID Frailty framework [34], which emphasizes Activity, Vaccination, Optimizing Medications, Interaction, and Diet. This framework targets key drivers of age-related decline and aligns closely with established management strategies for the non-surgical management of osteoarthritis [35,36]. Physical activity remains a cornerstone of frailty management [37,38], with regular strength, balance, and aerobic exercise shown to improve muscle mass, mobility, and overall function, thereby reducing frailty risk and enhancing resilience to aging-related conditions [37,39,40]. Vaccination plays a protective role by reducing susceptibility to infections that can accelerate frailty and lead to hospitalization [41], with evidence demonstrating substantial reductions in mortality among vaccinated older adults [42]. Optimizing medications is also critical, as polypharmacy is associated with increased risks of falls [43], cognitive impairment, and further functional decline; routine medication reviews can help minimize these risks and support independence [44]. Social interaction is another key component, as strong social connections are associated with better health outcomes, while loneliness and isolation increase vulnerability to frailty [45]. Finally, maintaining a healthy diet supports muscle preservation, cognitive function, and energy levels, all of which are essential for mitigating frailty [46,47]. Collectively, these interconnected strategies provide a comprehensive and clinically relevant approach to managing frailty and promoting healthy aging.

This study has several strengths. The use of a large, well-characterized cohort with repeated measures of frailty allows for robust, observational analyses. The time-varying Cox approach provides a nuanced understanding of how changes in frailty impact osteoarthritis risk, which is more informative than models relying solely on baseline measurements. However, there are limitations. Osteoarthritis status was based on participant self-reporting, which may be subject to misclassification; however, results were consistent with previous literature suggesting that potential misclassification had limited effect. Additionally, the wording of osteoarthritis diagnosis (“ever” diagnosed), prevented the precise timing of osteoarthritis onset between assessment waves, and may miss individuals with early osteoarthritis phenotypes who have not yet entered the healthcare system. Given the limited number of direct osteoarthritis related outcomes within the CLSA and the exclusion of variables used in the construction of the frailty index from regression models, the analyses may be subject to residual confounding. Due to the homogeneity of the dataset, with 95% of participants identifying as white, we are unable to examine the influence of race in this analysis. Factors that adjust the frailty-osteoarthritis relation warrant further investigation.

In conclusion, our study supports that longitudinal changes in frailty are associated with incident lower-limb osteoarthritis in both males and females over six-years. Changes in frailty over time further increase osteoarthritis risk, highlighting the importance of dynamic health monitoring. Notably, these associations were consistent regardless of sex and frailty measurement tool. These findings support the integration of frailty assessment into clinical practice and suggest that interventions targeting frailty may offer a novel avenue for osteoarthritis prevention and risk reduction.

## Author contribution

All authors were responsible for the conceptualization of the project. CH was responsible for all statistical analyses. CH completed the initial manuscript draft. RM and MWO were responsible for supervision. All authors were contributed to manuscript edits, refinement and approve the final version of the article. MWO (myles.obrien@usherbrooke.ca) takes responsibility for the integrity of the work as a whole, from inception to finished article.

## Data sharing

Data are available from the Canadian Longitudinal Study on Aging (www.clsa-elcv.ca) for researchers who meet the criteria for access to de-identified CLSA data.

## Disclaimers

The opinions expressed in this article are the author's own and do not reflect the views of the CLSA.

## Funding

This work was supported by the Canadian Institute of Health Research Catalyst Grant (award number: 539914); Carson Halliwell is funded by Arthritis Society Canada Postdoctoral Fellowship Salary Award (TPF) 25–0000000157. Funders had no say in study design, development or manuscript generation.

## Conflicts of interest

MWO is the Director of the Sedentary Behaviour Research Network. Sedentary Behaviour Research Network had no influence on the study results.
