# Supplementary material for: Frailty is associated with lower-limb osteoarthritis incidence over six-years regardless of sex and type of frailty index in the Canadian longitudinal study on aging
Source: Osteoarthr Cartil Open. 2026 May 27;8(3):100827. doi: 10.1016/j.ocarto.2026.100827 (PMC13251491; doi:10.1016/j.ocarto.2026.100827)
Supplement: Multimedia component 1 [file mmc1.docx]

**Supplemental Table 1**. Items included in self-report frailty index

| SELF REPORT FRAILTY INDEX (46 ITEMS) | |
| --- | --- |
| **Chronic Conditions** | **Self-rated health** |
| 1. Chronic Obstructive Pulmonary disease | 29. General health |
| 1. High blood pressure | 30. Vision |
| 1. Diabetes mellitus | 31. Hearing |
| 1. Chronic heart failure | **Activities of daily living** |
| 1. Angina | 32. Dressing |
| 1. Acute myocardial infarction | 33. Grooming |
| 1. Peripheral vascular disease | 34. Walking |
| 1. Stroke | 35. Getting in/out of bed |
| 1. Transient ischemic attack | 36. Bathing |
| 1. Memory problem | **Instrumental activities of daily living** |
| 1. Alzheimer’s disease | 37. Using the phone |
| 1. Parkinson’s disease | 38. Using transport |
| 1. Peptic ulcer disease | 39. Shopping |
| 1. Colitis | 40. Cooking |
| 1. Bowel incontinence | 41. Doing housework |
| 1. Urinary incontinency | 42. Taking medicine |
| 1. Cataracts | 43. Managing money |
| 1. Glaucoma | **Mental Health** |
| 1. Macular degeneration | 44. Effort |
| 1. Cancer | 45. Felt lonely |
| 1. Back pain | 46. Could not get going |
| 1. Hypothyroidism |  |
| 1. Hyperthyroidism |  |
| 1. Kidney failure |  |
| 1. Pneumonia |  |
| 1. Urinary tract infection |  |
| 1. Osteoporosis |  |
| 1. Falls |  |
