# Supplementary material for: Frailty is associated with lower-limb osteoarthritis incidence over six-years regardless of sex and type of frailty index in the Canadian longitudinal study on aging
Source: Osteoarthr Cartil Open. 2026 May 27;8(3):100827. doi: 10.1016/j.ocarto.2026.100827 (PMC13251491; doi:10.1016/j.ocarto.2026.100827)
Supplement: Multimedia component 2 [file mmc2.docx]

**Supplemental Table 2**. Items included in comprehensive frailty index

| FI-SELF REPORT (46 ITEMS) | | FI-EXAMINATION (40 ITEMS) | |
| --- | --- | --- | --- |
| **Chronic Conditions** | **Self-rated health** | **Physical Performance** | **Cardiac** |
| 1. Chronic Obstructive Pulmonary disease | 29. General health | 47. Chair Stand | 72. Systolic BP |
| 1. High blood pressure | 30. Vision | 48. Timed up and go | 73. Diastolic BP |
| 1. Diabetes mellitus | 31. Hearing | 49. Standing Balance  50. Grip strength | 74. Pulse |
| 1. Chronic heart failure | **Activities of daily living** | **Cognition** | 75. Pulse pressure |
| 1. Angina | 32. Dressing | 51. Immediate recall | 76. Average cardiac intima thickness, right |
| 1. Acute myocardial infarction | 33. Grooming | 52. Delayed recall | 77. Average cardiac intima thickness, left |
| 1. Peripheral vascular disease | 34. Walking | 53. Mental Alteration Test | 78. Presence of plaques |
| 1. Stroke | 35. Getting in/out of bed | 54. Animal fluency | 79. ECG diagnosis summary |
| 1. Transient ischemic attack | 36. Bathing | 55. Controlled Oral Word Association | 80. ECG, PQ interval |
| 1. Memory problem | **Instrumental activities of daily living** | 56. Time-based memory | 81. ECG, QRS duration |
| 1. Alzheimer’s disease | 37. Using the phone | 57. Event-based Memory | 82. ECG, QT interval |
| 1. Parkinson’s disease | 38. Using transport | 58. Choice reaction time | 83. ECG, P axis |
| 1. Peptic ulcer disease | 39. Shopping | 59. Stroop interference time | 84. ECG, R axis |
| 1. Colitis | 40. Cooking | **Anthropometric measures** | 85. ECG, T axis |
| 1. Bowel incontinence | 41. Doing housework |  | 86. ECG, P duration |
| 1. Urinary incontinency | 42. Taking medicine | 60. Waist to Hip Ratio |  |
| 1. Cataracts | 43. Managing money | **Spirometry** |  |
| 1. Glaucoma | **Mental Health** | 61. Forced Vital Capacity (FVC) |  |
| 1. Macular degeneration | 44. Effort | 62. Forced Expiratory Volume 1/FVC Ratio |  |
| 1. Cancer | 45. Felt lonely | **Hearing and Vision** |  |
| 1. Back pain | 46. Could not get going | 63. Visual acuity, left eye |  |
| 1. Hypothyroidism |  | 64. visual acuity, right eye |  |
| 1. Hyperthyroidism |  | 65. intraocular pressure, left |  |
| 1. Kidney failure |  | 66. intraocular pressure, right |  |
| 1. Pneumonia |  | 67. Corneal hysteresis, left |  |
| 1. Urinary tract infection |  | 68. Corneal hysteresis, right |  |
| 1. Osteoporosis |  | 69. Mean ocular perfusion pressure |  |
| 1. Falls |  | 70. Hearing pure tone average, left |  |
|  |  | 71. Hearing pure tone average, right |  |
