# Supplementary material for: Frailty is associated with lower-limb osteoarthritis incidence over six-years regardless of sex and type of frailty index in the Canadian longitudinal study on aging
Source: Osteoarthr Cartil Open. 2026 May 27;8(3):100827. doi: 10.1016/j.ocarto.2026.100827 (PMC13251491; doi:10.1016/j.ocarto.2026.100827)
Supplement: Multimedia component 3 [file mmc3.docx]

**Supplemental Table 3.** Cox regression output for knee and hip osteoarthritis separately.

|  | *Males* | *Females* |
| --- | --- | --- |
| ***Self-Report Frailty*** |  |  |
| Knee Osteoarthritis | 1.047 [1.041: 1.052] | 1.051 [1.047: 1.055] |
| Hip Osteoarthritis | 1.060 [1.052: 1.067] | 1.064 [1.059: 1.070] |
| ***Comprehensive Frailty*** |  |  |
| Knee Osteoarthritis | 1.054 [1.045: 1.063] | 1.058 [1.051:1.065] |
| Hip Osteoarthritis | 1.058 [1.046:1.071] | 1.052 [1.044: 1.061] |

Values represent hazard ratios and [95% confidence intervals], representing the risk of hip and knee osteoarthritis development for every 0.01-point increase in frailty.
