# Supplementary material for: Frailty is associated with lower-limb osteoarthritis incidence over six-years regardless of sex and type of frailty index in the Canadian longitudinal study on aging
Source: Osteoarthr Cartil Open. 2026 May 27;8(3):100827. doi: 10.1016/j.ocarto.2026.100827 (PMC13251491; doi:10.1016/j.ocarto.2026.100827)
Supplement: Multimedia component 4 [file mmc4.docx]

**Supplemental Table 4.** Cox regression results from sensitivity analyses restricting the self-reported frailty index sample to participants with available comprehensive frailty index data.

| **Self-Reported Frailty** | *Pooled Sample* | *Males* | *Females* |
| --- | --- | --- | --- |
| Sex | 0.718 [0.659: 0.782] | - | - |
| Frailty×sex | 0.995 [0.988: 1.002] | - | - |
| Frailty index | 1.051 [1.046: 1.056] | 1.047 [1.040: 1.054] | 1.051 [1.046: 1.056] |
| Age (years) | 1.022 [1.020: 1.025] | 1.021 [1.017: 1.026] | 1.023 [1.020: 1.027] |
| BMI (kg/m^2^) | 1.001 [1.001: 1.002] | 1.003 [1.002: 1.004] | 1.001 [1.000: 1.002] |
| Married, no. of (%) | 1.000 [ 1.000: 1.000] | 0.940 [0.892: 0.990] | 1.000 [1.000: 1.000] |

Values represent hazard ratios and [95% confidence intervals], representing the risk of lower-limb osteoarthritis development for every 0.01-point increase in frailty.
